# A cohort study of the bacterial faecal microbiota development post-weaning in healthy piglets in pig herds with different intestinal health status

**DOI:** 10.1186/s12866-026-05888-7

**Published:** 2026-11-06

**Authors:** Mikaela Klahr Fritz, Thomas Rosendal, Mikael Leijon, Axel Sannö, Per Wallgren, Marie Sjölund

**Affiliations:** 1 Department of Clinical Sciences, Swedish University of Agricultural Sciences, SE-750 07, Uppsala, Sweden; 2 Swedish Veterinary Agency (SVA), SE-751 89, Uppsala, Sweden

**Keywords:** Gastrointestinal, Gut, Porcine, Dysbiosis, Zinc-oxide, Diarrhoea, Zinc, Weaning

## Abstract

**Background:**

Weaning of piglets in commercial production involves a sudden separation from the sow and an abrupt switch to a grain-based feed. During this phase of gut microbiota adaptation, disease-associated bacteria, such as enterotoxin-producing *Escherichia coli*, can proliferate, causing post-weaning diarrhoea (PWD). An observational cohort study of the faecal microbiota development during the first three weeks post-weaning in apparently healthy piglets from three farms was conducted. Four sample series were made, where Farm 1, which had no clinical signs of PWD, was sampled twice. Farm 2 had intermittent PWD problems, and Farm 3 used high-dose zinc oxide (ZnO) after weaning. Rectal swab samples were collected before weaning and on days 3, 7, 10, 14 and 21 after weaning. The bacterial faecal microbiota was assessed using 16S rRNA V3-V4 variable region sequencing.

**Results:**

Of the identified amplicon sequence variants, 99% and 84% could be classified at the phylum and family level, respectively. Firmicutes and Bacteroidota were the two dominant phyla in all study groups. In Farm 2, with intermittent PWD problems, the relative abundance of Proteobacteria was significantly higher compared to the other study groups (*P* < 0.05). There was a significantly higher relative abundance of bacteria from the family Prevotellaceae in the second study group from Farm 1. The alpha diversity increased post-weaning in all study groups except for Farm 3, which used ZnO. Beta diversity showed an increased similarity between the piglets’ microbiota over time and across farms.

**Conclusions:**

A difference between the gut microbiota of piglets before and after weaning was found in all study groups. Weaning led to an imbalance in gut microbiota and changes to the faecal consistency, even in piglets without clinical PWD. The faecal microbiota composition differed between study groups and across the study period, also between repeated samplings of the same farm. The higher relative abundance of Proteobacteria in the herd with intermittent PWD problems could be a contributing factor to the recurring issues with PWD in this herd. The use of ZnO seemed to have delayed the maturation of the microbiota, although further studies are needed to confirm this. Overall, the results show that no single microbiota composition can predict weaning without gut disturbances.

**Supplementary information:**

The online version contains supplementary material available at https://doi.org/10.1186/s12866-026-05888-7.

## Background

Free-ranging piglets are fully weaned at an average age of approximately 17 weeks, after a gradual process where the piglet and its gut progressively adapt to solid feed [1, 2]. In contrast, weaning in commercial indoor pig production involves a sudden separation from the sow, typically at three to six weeks of age, but may take place as early as two weeks of age [3]. The abrupt switch to a grain-based feed, as well as other stressors associated with this practice, have adverse effects on piglet gut health [4–6]. The permeability of the intestinal barrier increases post-weaning and is accompanied by a reduction in villus height and villus-to-crypt ratio, especially in early-weaned pigs. These changes induced by weaning result in decreased nutritional uptake [7–10]. Moreover, the piglets’ adaptive immunity is poorly developed, and their maternal immunity is declining at the time of weaning in commercial pig production [11]. In addition, the piglets no longer get IgA antibodies from the sow milk, which also increases their susceptibility to intestinal disease [12].

The intestinal microbiota of pigs is normally dominated by bacteria from the phyla Firmicutes and Bacteroidota across all ages [13]. The changes at weaning affect the gut microbiota, where bacteria adapted to milk no longer thrive, and the altered conditions favour bacteria better adapted to a grain-based feed. During this transition period, dysbiosis can occur, enabling disease-associated bacteria to proliferate. Post-weaning diarrhoea (PWD) may arise when enterotoxin-producing *Escherichia coli* (ETEC) bacteria, belonging to the phylum Proteobacteria, proliferate, adhere to specific receptors on mucosal epithelial cells, and produce enterotoxins [14, 15]. Clinical signs of PWD are typically observed around one week after weaning and can vary in severity. Common characteristics include diarrhoea, reduced growth, and dehydration, generally causing an increased mortality rate [16, 17]. Thus, PWD causes ill-thrift in affected piglets, increases the need for antibiotic treatments as well as the workload, leading to financial losses. These losses are estimated to range from €33 to €250 per sow and year [18]. Prevention of PWD is challenging because of its multifactorial aetiology, dependent on genetic susceptibility, colostrum intake, feed composition, feed intake, management, and environmental factors such as temperature and hygiene [19].

When the gut microbiota adapts to the new conditions after weaning, an increase in the family Prevotellaceae and genus *Prevotella* is seen, as these bacteria can digest plant polysaccharides [13, 20–22]. Alpha diversity indices (including richness) generally increase after weaning [22–24], whereas the diversity of coliform bacteria decreases [25]. Spirochaetota generally shows a transient increase after weaning, mostly represented by genus *Treponema*, which has been identified as a potential biomarker of gut microbiota associated with diarrhoea in piglets [26]. This phylum also includes bacterial taxa associated with enteric disease post-weaning, such as the genus *Brachyspira* [27]. The phylum Desulfobacterota may also show a transient increase in relative abundance post-weaning [28]. *Lawsonia intracellularis (L. intracellularis)*, causing proliferative enteropathy, is one pathogenic species within this phylum [29]. These bacteria can contribute to gastrointestinal disease after weaning, although primarily later post-weaning.

Supplementation of high-dose dietary zinc oxide, hereafter referred to as ZnO, for the first 14 days after weaning can be used to prevent PWD in piglets [30]. The intestinal coliform microbiota is stabilised by ZnO at weaning without reducing the number of excreted *Escherichia coli* (*E. coli*) [25, 31, 32]. Studies using biochemical fingerprinting have shown that the diversity of the coliform intestinal flora was higher after weaning in piglets fed a ZnO feed than in untreated piglets [25, 33]. Later studies have shown that ZnO appears to suppress Gram-positive commensals such as lactic acid bacteria and favour diversity among Gram-negative coliform bacteria such as *E. coli* [34]. Zinc oxide has also been shown to protect enterocytes from damage caused by ETEC and seems to have an immunomodulatory effect [35, 36]. Before the ban of ZnO in the European Union (EU) in June 2022, ZnO was frequently prescribed for the prevention of PWD in herds with recurring gastrointestinal health issues after weaning. The ban was introduced due to the potential environmental impact [37, 38]. After the ban, the sales of antibiotic substances commonly used for treating PWD: neomycin, paromomycin, and colistin have increased, which reflects an escalated problem with PWD [39, 40]. The increased use of antibiotics promotes the development of antibiotic resistance, which could make treatment of PWD more challenging or impossible in the future. This would substantially impact pig health, animal welfare, as well as the economy of pig producers.

This observational cohort study aimed to investigate the development of the intestinal microbiota in healthy piglets during the post-weaning period from selected herds with varying clinical and management conditions. The objectives were specifically to assess whether herd-level clinical status and ZnO treatment influence the microbial diversity and microbiota composition, and to characterise differences in microbial community development associated with herd-level clinical status and ZnO use.

## Methods

An observational cohort study of the faecal microbiota development during the first three weeks post-weaning in apparently healthy piglets from three herds was conducted. Rectal faecal swab samples were collected from a total of 32 pigs from three farms in mid-Sweden. Farm 1 was a herd without clinical PWD. Farm 2 was a herd with intermittent clinical PWD problems. Farm 3 used ZnO, and clinical signs of PWD were absent.

### Animals, experimental design and sample collection

In all herds, Yorkshire x Landrace sows were inseminated with Duroc or Hampshire semen. All farms vaccinated the sows against Parvovirus, erysipelas, and *E. coli* to prevent neonatal diarrhoea, and the sows were dewormed with avermectin ante partum. Batches of sows farrowed in previously emptied and cleaned farrowing units. Within the first three days of life, the piglets at all farms were injected with gleptoferron and toltrazuril to prevent iron deficiency and coccidiosis. All male piglets were surgically castrated under local anaesthesia and given a single intramuscular injection of a non-steroidal anti-inflammatory drug for pain alleviation. Neither tail docking, teeth clipping, nor teeth grinding was performed. All piglets were vaccinated against Porcine circovirus type 2 (PCV2) at an average age of three weeks. Neither prophylactic nor metaphylactic antibiotics were used. The piglets were weaned at a mean age of 32 days and transferred to emptied and cleaned weaning units. The weaner pens had a solid concrete floor (70%) with a slatted area (30%). All included farms practised age-segregated rearing, following an all-in/all-out management system.

Farm 1 was a 360-sow piglet-producing farm that had never experienced clinical PWD. The weaned piglets were fed commercial dry feed for weaners (Gottfrid, Lantmännen, Malmö, Sweden) with 15% crude protein and 10.1 net energy measured in megajoules per kilogram (NE MJ/kg). Wood shavings were used as bedding material for the weaned piglets, with daily straw allowances provided for enrichment. This study group is referred to as Farm 1/set-a (PWD-, ZnO-). A second study group, referred to as Farm 1/set-b (PWD-, ZnO-), from Farm 1 was also included in the study. These samples were collected three months after the first sampling to study the variation over time of the intestinal microbiota in pigs in a herd unaffected by PWD. The pigs in the second study group were also fed Gottfrid, however, from another manufacturing batch.

Farm 2 was a piglet-producing farm within a multisite production system where five groups of 40 sows farrowed during a ten-week cycle, corresponding to a herd size of 380 sows. The sows farrowed in cycles of every second week for 6 weeks, and thereafter 4 weeks without any farrowings. This herd was intermittently affected by PWD. Wood shavings were used as bedding material for the first day after weaning, followed by straw for the subsequent period. The weaned piglets were fed a liquid diet composed of commercial concentrate and farm-produced grains, providing 15.6% crude protein and 9 NE MJ/kg of dry matter, with an additional meal of dry feed offered daily during the first week after weaning. This study group is referred to as Farm 2 (PWD + , ZnO-).

Farm 3 was a 750-sow farrow-to-finish farm without clinical PWD, in which dietary ZnO was used in weaners to prevent PWD. The samples were collected before the ban on ZnO was enforced. In this herd, the piglets were also vaccinated against *L. intracellularis* and *Mesomycoplasma hyopneumoniae* at approximately three weeks of age, and against *Actinobacillus pleuropneumoniae* at approximately six weeks of age. The weaned piglets were fed a commercial dry feed (Zigge Max, Lantmännen, Malmö, Sweden) containing ZnO (2500 ppm), crude protein 16% and energy 9.4 NE MJ/kg, which was mixed with water for the first two weeks after weaning and served as a liquid feed. Thereafter, the pigs were fed a liquid feed with commercial concentrate and farm-produced grains. Wood shavings were used as bedding material for the weaned piglets, with daily straw allowances for enrichment. This study group is referred to as Farm 3 (PWD-, ZnO + ).

Before weaning, one apparently healthy, average-sized piglet from each of four different litters in a farrowing batch was given an identity. This was repeated in the consecutive farrowing batch. Thus, each study group included eight weaners from two consecutive farrowing batches. Individual faecal swab samples were collected before weaning and on days 3, 7, 10, 14, and 21 post-weaning. In conjunction with sampling, the faecal consistency was recorded for each sampled piglet and classified using three categories: normal, loose, and diarrhoea. Normal faeces were firm to soft and shaped, whereas loose faeces were poorly formed while retaining some visible structure. Faeces scored as diarrhoea were liquid and lacked formed material. If a pig developed any clinical disease requiring treatment, it was replaced with an average-sized littermate. Hence, no pig included in the study had been treated with antibiotics.

### Genomic DNA extraction and purification

Faecal swab samples were collected using E-swabs® (Copan Italia S.p.A., Italy) inserted 2-4 cm into the rectum of the piglets and rotated before extraction. The samples were kept at -20 °C from sampling until analysis. After thawing, the samples were vigorously vortexed to release the faecal material from the swab into the liquid and centrifuged at 3000×g for 10 minutes. 120 µl of the pelleted material was dissolved in 1 ml ASL buffer (QIAGEN, Venlo, The Netherlands) and homogenized with 2 mm zirconia beads using FastPrep-24 Sample Preparation System (MP Biomedicals, Irvine, CA, USA) with the setting 6.5 m/s for 2 × 60 s, incubated at 95 °C for 5 min, followed by centrifugation at 14,000 × g for 1 min. Two hundred µl of the supernatant was mixed with 20 µl proteinase K, and DNA was extracted with IndiMag®Pathogen kit (Indical Bioscience GmbH, Leipzig, Germany).

### Sequencing and bioinformatic data analysis

The samples were divided into two Next-Generation Sequencing (NGS) runs with 96 samples in each, with one run dedicated to samples from Farm 1/set-a and Farm 1/set-b, and the other to the samples from Farm 2 and Farm 3. A 16S amplicon library, targeting V3–V4 hypervariable regions, was prepared according to the Illumina 16S Metagenomic Sequencing Library Preparation protocol (Illumina Inc., San Diego, CA, USA). After validation and quantification on Agilent 2100 Bioanalyzer (Agilent, Santa Clara, CA, USA) and Qubit (Thermo Fisher Scientific Inc., Waltham, MA, USA), the libraries were combined at equimolar concentrations, denatured, and diluted to 8 pM. A 10% PhiX library control was spiked. Paired-end amplicon sequencing was thereafter conducted on an Illumina MiSeq sequencer using a 2×300-cycle MiSeq reagent v3 kit (Illumina Inc., San Diego, CA, USA).

Before further data processing, six samples with the number of reads below 30% of the median number of reads across all samples in the run were excluded to avoid samples with insufficient sampling of the microbiota. The amplicon sequence variants (ASVs) method was employed to analyse the amplicon sequence data [41]. The sequence reads were processed using the Divisive Amplicon Denoising Algorithm 2 (DADA2) software version 1.24 [42]. With DADA2, error rates and sample inference were calculated for each run separately, after which the runs were merged, chimaeras were removed, and taxonomies were assigned. Taxonomies were based on SILVA version 138.1 [43]. DADA2 was run with the following parameters: truncation length of the reads (truncLen) was 280 and 260 nucleotides (nt) for forward and reverse reads, respectively; the minimum read length (minLen) was 200 nt; trimleft was set to 17 and 30, for forward and reverse reads, respectively, and served to remove primer sequences; the maxEE parameter was disabled by setting it to Inf, and truncQ was set to 2. In addition, all amplicons shorter than 300 nt were removed.

### Diversity, visualisation and statistical analysis

Data from the DADA2 pipeline were further processed and visualised in R (version 4.6.1) using the phyloseq package (version 1.56.0) [44]. Summaries of abundance among phylogenetic classifications across farms from individual pigs were logit-transformed, and 95% confidence intervals were calculated by 1.96 × the standard error of the sample abundances within a grouping and all ranges in the results section have been determined like this. Associations of abundance of taxonomic classes with study group and the before-weaning to post-weaning period were tested using a logistic regression model including a random effect to adjust for the repeated measures of the same pigs within each study group over time and a fixed effect for the study groups. Samples collected on day-0 were classified as before weaning and all other samples were post-weaning. These models were implemented in R (version 4.6.1) using the lme4 package (2.0-6) [45]. Rarefaction was performed at a depth of 18,484 reads, corresponding to the minimum number of reads among the samples. As rarefaction had no apparent effect on the alpha- or beta-diversity measures, the results presented are based on non-rarefied data. The Shannon diversity index was used to summarise the alpha diversity among the samples, and the results were visualised using the R package phyloseq (version 1.56.0). Beta diversity among samples was computed using the Bray–Curtis dissimilarity and visualised using non-metric multidimensional scaling (NMDS) to visualise the samples (phyloseq version 1.56.0). To examine differences in microbial community composition between study groups and days after weaning, permutational multivariate analysis of variance (PERMANOVA) using Bray–Curtis dissimilarities was performed using the adonis2() function of the vegan (version 2.7-5) package [46]. Additionally, homogeneity of the dispersions was assessed using the betadisper() function in the vegan package to compare within-group variability in microbial community composition across study groups and days after weaning. Significance was tested using a permutation test with 999 permutations to evaluate differences in dispersion.

## Results

### Clinical observations

No diarrhoea was observed among the sampled pigs in Farm 1 (PWD-, ZnO-), but loose faeces were observed five times in study group 1a (in single pigs on days 7, 10, 14 and two pigs on day 21) and five times in study group 1b (in one single pig on day 3, two pigs on days 14 and 21). In total, two pigs at Farm 1 were replaced due to antibiotic treatment for non-enteric disease (one in the study group Farm 1/set-a and one in Farm 1/set-b).

At Farm 2 (PWD + , ZnO-), four sampled pigs had loose faeces on day 3, and one pig had diarrhoea. On day 7, one pig had loose faeces, and two had diarrhoea. On days 14 and 21, single pigs had loose faeces. No pig in the study group Farm 2 (PWD + , ZnO-) was replaced during the study and the observed diarrhoea resolved without treatment.

At Farm 3 (PWD-, ZnO + ), one pig included in the study had diarrhoea before weaning, one pig had loose faeces on day 10 after weaning, and two on day 21. No pig at Farm 3 (PWD-, ZnO + ) was replaced during the study.

### Farm-level shifts in the piglet faecal microbiota post-weaning

**Fig. 1:**
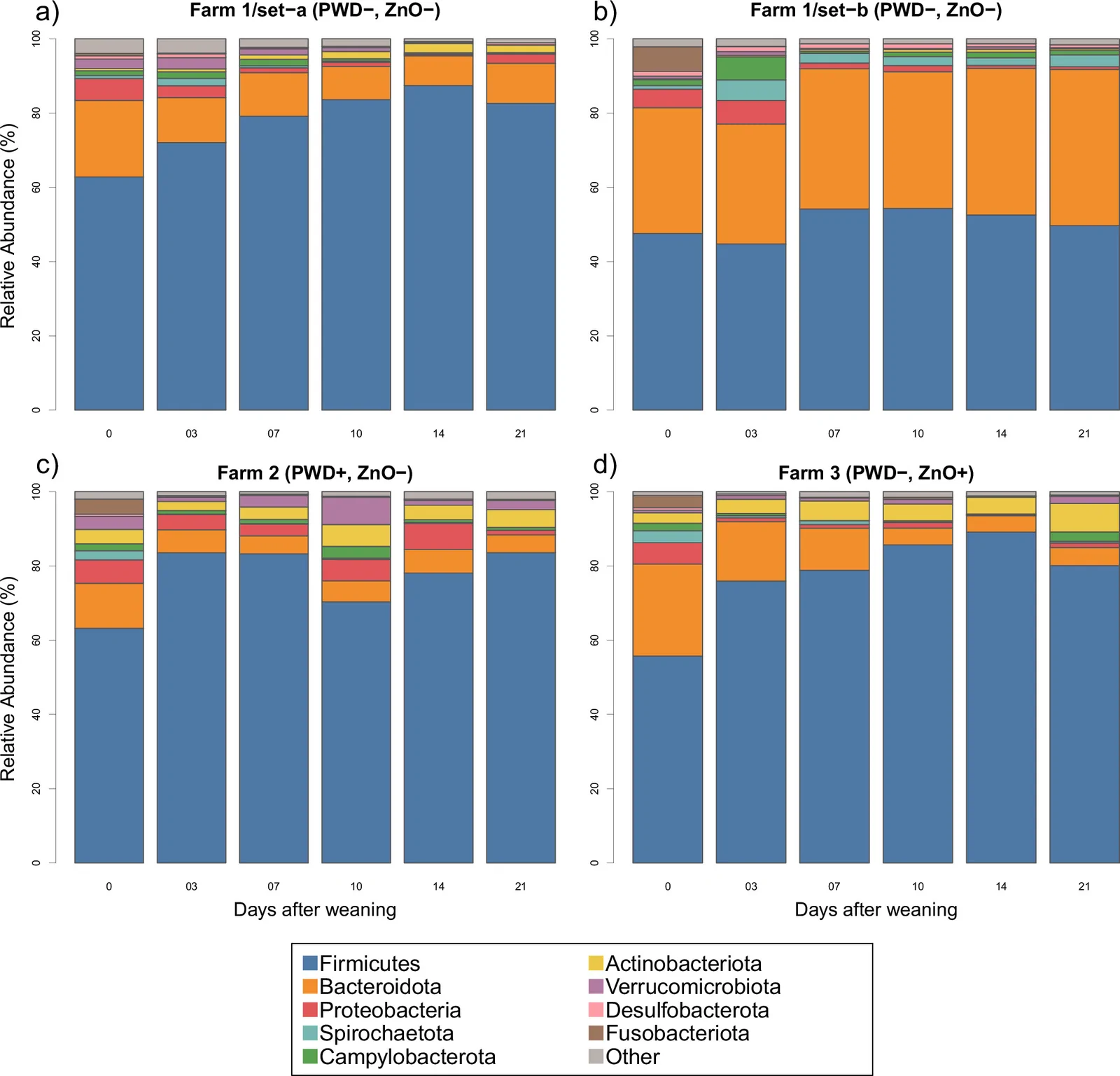
Average faecal microbiota composition during the post-weaning period at the phylum level in eight healthy piglets per panel, obtained by 16S rRNA gene amplicon sequencing of the V3-V4 regions. The relative abundance of the nine most prevalent phyla is shown in the figure, while the remaining phyla and uncategorised ASVs were grouped as “Other”. Panel (**a**) shows the farm without clinical post-weaning diarrhoea (Farm 1/set-a (PWD-, ZnO-)) and panel (**b**) shows the same farm three months later (Farm 1/set-b (PWD-, ZnO-)). Panel (**c**) shows the farm with intermittent problems with post-weaning diarrhoea (Farm 2 (PWD + , ZnO-)) and panel **d**) shows the farm using 2500 ppm of zinc oxide in the feed for 14 days after weaning (Farm 3 PWD-, ZnO + )

At the phylum level, 99% of the identified ASVs were classified. Across all study groups and time points, Firmicutes was the most abundant phylum, with a farm-level average relative abundance in the range of 0.45 - 0.89, and displayed a significant increase in all study groups post-weaning (*P* < 0.001) (Fig. 1). However, this increase was much less pronounced in set-b from Farm 1 (PWD-, ZnO-), with a relatively even distribution between the Firmicutes and Bacteroidota phyla (Fig. 1). In addition, in set-b from Farm 1 (PWD-, ZnO-), the average relative abundance of Bacteroidota slightly increased after weaning (Fig. 1), opposite to the trend in the other three study groups, where the relative abundance significantly decreased after weaning (*P* < 0.001).

The relative abundance of bacteria from the phylum Proteobacteria was significantly higher in Farm 2 (PWD + , ZnO-) compared to the other study groups (*P* < 0.05). This difference was particularly pronounced on days 10 and 14 (Fig. 1). In all study groups, the relative abundance of Proteobacteria decreased significantly post-weaning (*P* < 0.001). A relatively high abundance of Actinobacteriota was observed in the samples from Farm 2 (PWD + , ZnO-) and Farm 3 (PWD-, ZnO + ), with a combined geometric mean of 0.04 (0.00, 0.15). The combined geometric mean for Farm 1/set-a (PWD-, ZnO-) and Farm 1/set-b (PWD-, ZnO-) was 0.01 (0.00, 0.05), which differed significantly (*P* < 0.001) from Farm 2 (PWD + , ZnO-) and Farm 3 (PWD-, ZnO + ). In contrast to before weaning, and to the post-weaning period in both study groups from Farm 1 (PWD-, ZnO-), Actinobacteriota was the second most abundant phylum at day 21 on Farm 2 (PWD + , ZnO-) and Farm 3 (PWD-, ZnO + ) (Fig. 1).

Pigs in Farm 2 (PWD + , ZnO-) had a significantly (*P* < 0.001) higher relative abundance of bacteria from the phylum Verrucomicrobiota (0.01 (0.00, 0.27)) when compared to the average of all other study groups (0.002 (0.000, 0.047]). In fact, Verrucomicrobiota was the second-most abundant phylum on day 10 at Farm 2 (PWD + , ZnO-) (Fig. 1). Bacteria from the phylum Fusobacteriota were present in the microbiota before weaning in all study groups (0.01 (0.00, 0.38)) but then decreased in relative abundance during the post-weaning phase in all groups (0.0001 (0.0000, 0.0036]. Bacteria classified to the Fusobacteriota phylum were absent in 47/157 postweaning samples (Fig. 1). Throughout the sampling period, the relative abundance of Spirochaetota in Farm 1/set-b (PWD -​, ZnO-) was significantly higher (0.02 (0.01, 0.02]; *P* < 0.001) compared to the other study groups (0.001 (0.001, 0.002)). Spirochaetota was detected in all samples except one across the other study groups. Desulfobacterota showed a similar pattern, but with a lower relative abundance (Fig. 1). A low relative abundance of bacteria from the phylum Campylobacterota was observed throughout the sampling period in all study groups. However, on day 3 at Farm 1/set-b (PWD-, ZnO-), one pig had a transient relative abundance of 43% of the phylum Campylobacterota, of which Helicobacteriaeceae was the dominant family (Supplementary material Fig. 1).

**Fig. 2:**
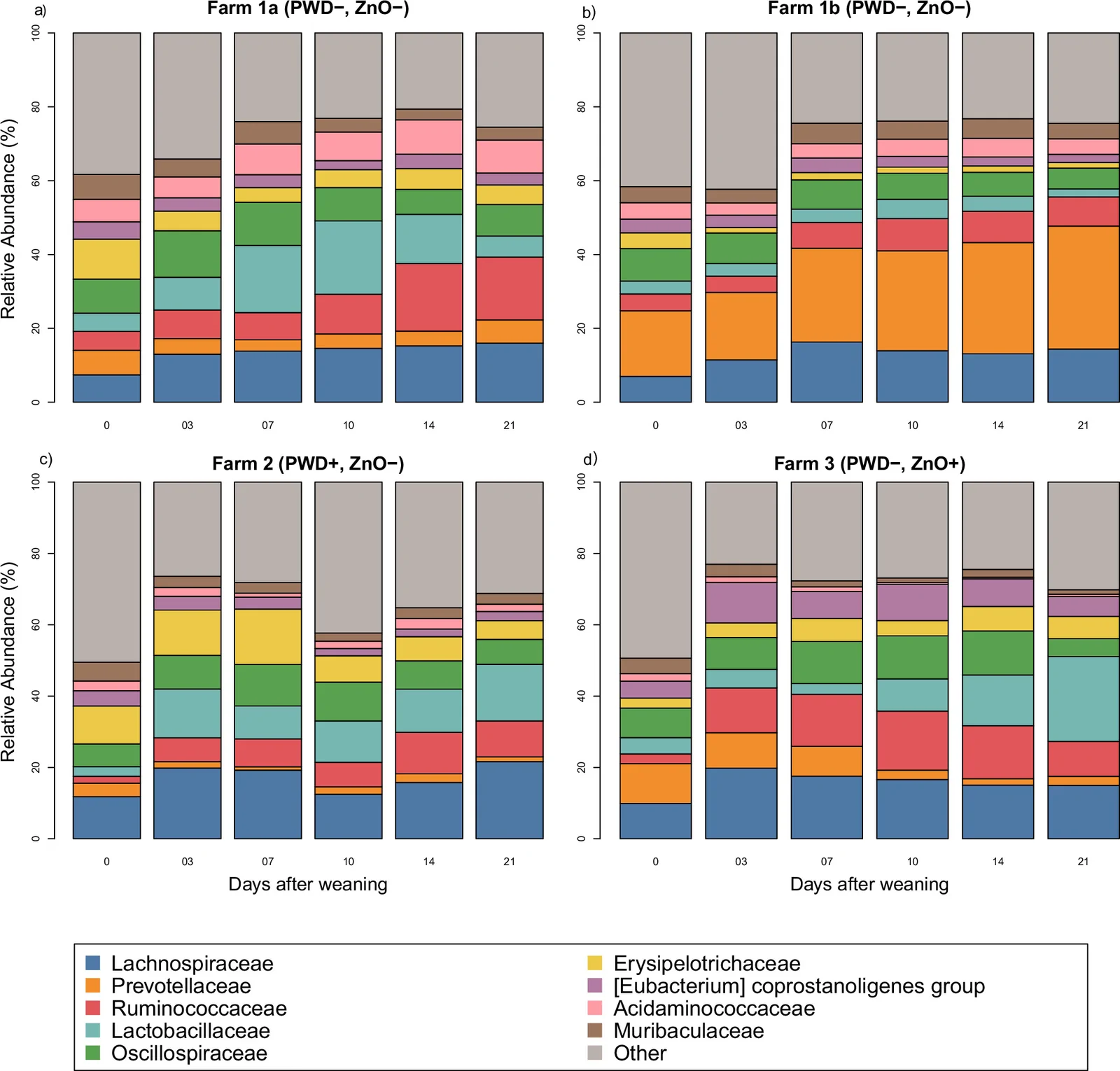
Average faecal microbiota composition during the post-weaning period at the family level in eight healthy piglets per panel, obtained by 16S rRNA gene amplicon sequencing of the V3-V4 regions. The relative abundance of the nine most prevalent families is shown in the figure, while the remaining families and uncategorised ASVs were grouped as “Other”. Panel **a**) shows the farm without clinical post-weaning diarrhoea (Farm 1/set-a PWD-, ZnO-) and panel **b**) shows the same farm three months later (Farm 1/set-b). Panel **c**) shows the farm with intermittent problems with post-weaning diarrhoea (Farm 2 PWD + , ZnO-) and panel **d**) shows the farm using 2500 ppm of zinc oxide in the feed for 14 days after weaning (Farm 3 PWD-, ZnO + )

At the family level, 84% of the identified ASVs could be classified. Figure 2 displays the composition of the faecal microbiota at the taxonomic family level across the four study groups, with the nine most abundant families displayed. Seven of these families belonged to the phylum Firmicutes, namely: Acidaminococcaceae, *Eubacterium coprostanoligenes* group, Erysipelotrichaceae, Lachnospiraceae, Lactobacillaceae, Oscillospiraceae, and Ruminococcaceae. The two remaining families, Muribaculaceae and Prevotellaceae, belonged to the phylum Bacteroidota. The combined average relative abundance of these nine families increased in all study groups during the post-weaning period, from 0.55 to 0.74 (Fig. 2).

For Farm 1/set-a (PWD-, ZnO-), the increase mainly consisted of Lachnospiraceae and Ruminococcaceae, while in Farm 1/set-b (PWD-, ZnO-), it mainly consisted of Prevotellaceae (Fig. 2). The latter observation parallels the increase in the relative abundance of the phylum Bacteroidota seen for this group (Fig. 1). However, Prevotellaceae either decreased or remained fairly constant at much lower abundance in the other study groups. In Farm 2 (PWD + , ZnO-) and Farm 3 (PWD-, ZnO + ), Lactobacillaceae, Lachnospiraceae, and Ruminococcaceae were the families that mainly constituted the increase. Transient increases were seen for Lactobacillaceae, Erysipelotrichaceae, and *Eubactrium coprostanoligenes* group in Farm 1/set-a (PWD-, ZnO-), Farm 2 (PWD + , ZnO-), and Farm 3 (PWD-, ZnO + ), respectively during the study period (Fig. 2). The relative abundance of bacteria from the family Erysipelotrichaceae was significantly higher in Farm 2 (PWD + , ZnO-) compared to the other study groups (*P* < 0.001), which was most prominent on days three and seven after weaning (Fig. 2).

**Fig. 3:**
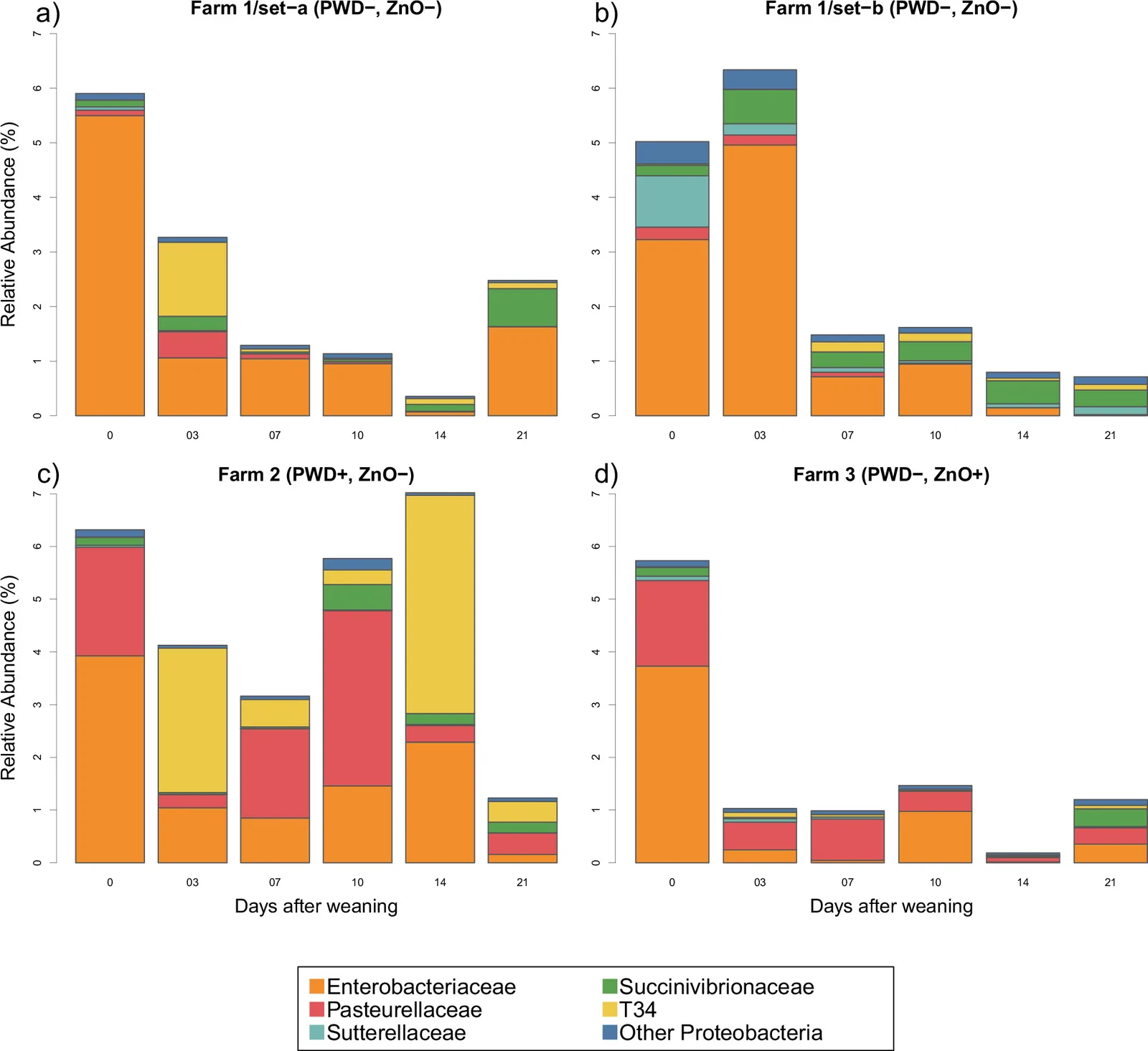
Average relative abundance of the four most common families, as well as the SILVA family group T34, within the phylum Proteobacteria in the faecal microbiota in eight healthy piglets per panel during the post-weaning period, obtained by 16S rRNA gene amplicon sequencing of the V3-V4 regions. The relative abundance of the four families and SILVA family group is shown in the figure, while the remaining ASVs classified at the phylum level as Proteobacteria were grouped as “Other”. Panel **a**) shows the farm without clinical post-weaning diarrhoea (Farm 1/set-a PWD-, ZnO-) and panel **b**) shows the same farm three months later (Farm 1/set-b). Panel **c**) shows the farm with intermittent problems with post-weaning diarrhoea (Farm 2 PWD + , ZnO-) and panel **d**) shows the farm using 2500 ppm of zinc oxide in the feed for 14 days after weaning (Farm 3 PWD-, ZnO + )

Figure 3 shows the relative abundance of the four most abundant Proteobacteria families: Enterobacteriaceae, Pasteurellaceae, Succinivibrionaceae, and Sutterellaceae, as well as the SILVA family group T34 (order Burkholderiales). The remaining ASVs classified as Proteobacteria are grouped under “Other Proteobacteria”. As seen at the phylum-level (Fig. 1), Proteobacteria generally decreased during the post-weaning period. However, as mentioned above, higher abundance levels of Proteobacteria were seen in the study group from Farm 2 (PWD + , ZnO-), especially on days 10 and 14. Proteobacteria family group T34 was more abundant on days 3 and 14 in this study group compared to the others. In both study groups from Farm 1 (PWD-, ZnO-), Enterobacteriaceae dominated the Proteobacteria phylum’s contribution to the piglet faecal microbiota, especially early during weaning. In addition to Enterobacteriaceae, Pasteurellaceae were abundant in samples from Farm 2 (PWD + , ZnO-) and Farm 3 (PWD-, ZnO + ). The relative abundance of bacteria from the Succinivibrionaceae family varied during the post-weaning period in all study groups. In Farm 1/set-b (PWD-, ZnO-), bacteria from the Sutterellaceae family were the second most abundant at weaning, after which their relative abundance decreased.

In Farm 1/set-a (PWD-, ZnO-), the mean relative abundance of the Bacteroidota family Prevotellaceae remained low throughout the study period (0.04 [0.01, 0.17)). Bacteria from the family Prevotellaceae also increased in Farm 1/set-b (PWD-, ZnO-) over time and were more abundant at all time points (0.24 [0.08, 0.53]), compared to the other study groups. In Farm 2 (PWD + , ZnO-), the mean relative abundance of Prevotellaceae was low (0.024 (0.001, 0.304)) at weaning and remained so throughout the studied post-weaning phase. In Farm 3 (PWD-, ZnO + ), the average relative abundance of Prevotellaceae at weaning was higher (0.095 [0.025, 0.304)) but had decreased to lower levels by day 10 after weaning (0.016 (0.002, 0.099]).

**Fig. 4:**
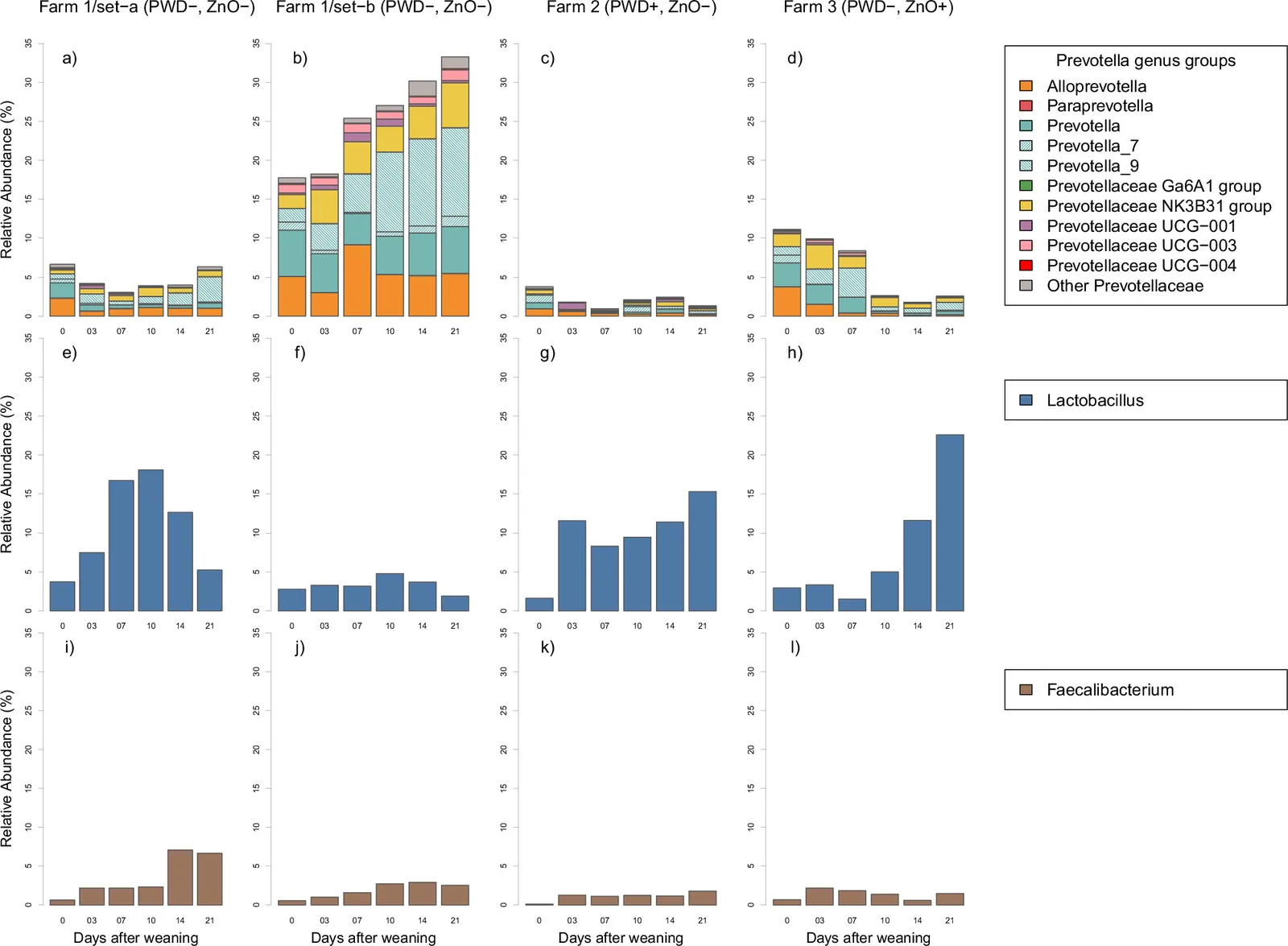
Average relative abundance of selected genera in the faecal microbiota of eight healthy piglets per panel during the post-weaning period, obtained by 16S rRNA gene amplicon sequencing of the V3-V4 regions. The first column (panels **a,e,i**) shows the farm without clinical post-weaning diarrhoea (Farm 1/set-a: PWD-, ZnO-) and the second column (panels **b,f,j**) shows the same farm three months later (Farm 1/set-b). The third column (panels **c,g,k**) shows the farm with intermittent problems with post-weaning diarrhoea (Farm 2 PWD + , ZnO-) and the fourth column (panels **d,h,l**) shows the farm using 2500 ppm of zinc oxide in the feed for 14 days after weaning (Farm 3 PWD-, ZnO + )

At the genus level, 62.5% of the identified ASVs were classified, but at the species level, only 2.1% of the identified ASVs were classified. The prevalence of the genera within the family Prevotellaceae is presented in panels a-d in Fig. 4. The relative abundance of the genus *Alloprevotella* (0.007 (0.000, 0.179)]) was fairly constant or decreased in the later time points, with a higher abundance in Farm 1/set-b (PWD-, ZnO-). In contrast, the average relative abundance of *Paraprevotella* was low in all study groups and time points. *Paraprevotella* was present in 42 of 186 samples with an average relative abundance of 0.0001 (0.0000, 0.0010) and is therefore not visible in Fig. 4. For the *Prevotella* genus, the V3-V4 16S sequencing data allowed the identification of the genus-level subgroups *Prevotella_7* and *Prevotella_9*. In both groups on Farm 1 (PWD-, ZnO-), the *Prevotella_9* group constituted the majority of the increase seen in *Prevotella*. In contrast, *Prevotella_9* decreased during the post-weaning period on the other farms.

Family-level clusters of hitherto uncultured bacteria, which represent potential novel genera have been discovered with NGS. Several of these, belonging to the *Prevotella* genus, were detected in the present study. The Prevotellaceae NK3B31 cluster increased after weaning in Farm 1/set-b (PWD-, ZnO-). In the other study groups, the Prevotellaceae NK3B31 cluster was present throughout the study period, but with a lower abundance. The Prevotellaceae uncultured subgroups Prevotellaceae Ga6A1, Prevotellaceae UCG-001, Prevotellaceae UCG-003, and Prevotellaceae UCG-004 were detected at low levels. However, in Farm 1/set-b (PWD-, ZnO-), the relative abundances of Prevotellaceae UCG-001 and Prevotellaceae UCG-003 were higher (Fig. 4).

The trends in the relative abundance of the *Lactobacillus* genus in the Firmicutes phylum during the monitored post-weaning period (middle row in Fig. 4) closely aligned with those of the Lactobacillaceae family (Fig. 2) across all farms. Although the pre-weaning mean relative abundance of *Lactobacillus* across all farms was low (0.015 (0.001, 0.150]), temporal changes during post-weaning varied between the study groups. On Farm 1/set-a (PWD-, ZnO-), there was a roughly fourfold increase by day 10, followed by a decline to near the pre-weaning level, while the abundance for Farm 1/set-b (PWD-, ZnO-) remained at low levels throughout the study period. In contrast, Farm 2 (PWD + , ZnO-) and Farm 3 (PWD-, ZnO + ) both showed an increase in relative abundance of *Lactobacillus* at day 21, to 0.15 [0.09, 0.25) and 0.19 [0.03, 0.64], respectively (Fig. 4).

The mean relative abundance of the genus *Faecalibacterium* in the Firmicutes phylum was low in all study groups (0.01 (0.00, 0.15)) except for Farm 1/set-a (PWD-, ZnO-), where it increased to 0.06 [0.03, 0.15) on average, at days 14 and 21 (bottom row in Fig. 4).

### The alpha diversity of the post-weaning microbiota

**Fig. 5:**
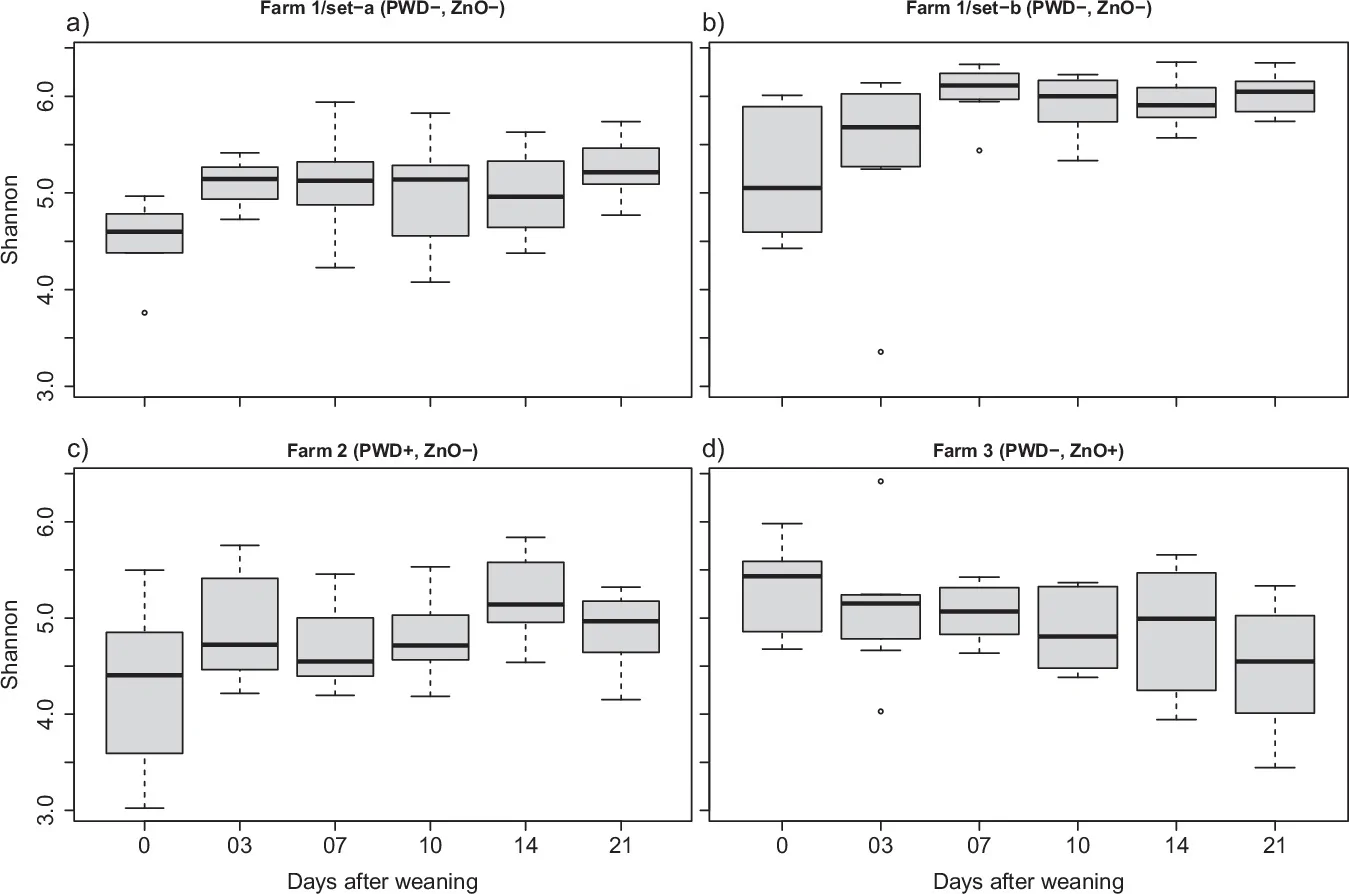
Temporal evolution of microbiota diversity (Shannon index) from pre-weaning to day 21 post-weaning in eight healthy piglets across three farms. Panels **a**) and **b**) each show the diversity at the farm without clinical post-weaning diarrhoea (PWD) collected three months apart (Farm 1/set-a and Farm 1/set-b). Panel **c**) shows the farm with intermittent problems with post-weaning diarrhoea (Farm 2 PWD + , ZnO-) and panel **d**) shows the farm using 2500 ppm of zinc oxide in the feed for 14 days after weaning (Farm 3 PWD-, ZnO + )

Figure 5 shows the development of population diversity in the piglet faecal microbiota as measured by the Shannon diversity index across the four study groups. The initial median diversity, before weaning, ranged from 4.4 in Farm 2 (PWD + , ZnO-) to 5.4 in Farm 3 (PWD-, ZnO + ), which represents nearly a threefold difference in the effective number of ASVs, assuming equal absolute abundance. Diversity increased in both study groups from Farm 1 (PWD-, ZnO-) and in Farm 2 (PWD + , ZnO-), while it decreased in Farm 3 (PWD-, ZnO + ). However, there was a difference between the two study groups from Farm 1, with a lower diversity throughout the study period in Farm 1/set-a (PWD-, ZnO-). Diversity after weaning was highest in Farm 1/set-b (PWD-, ZnO-) and had stabilised at 6.1 by day 7 post-weaning. In the other study groups, microbiota diversity among pigs varied more across the study period.

### Beta diversity analysis of post-weaning microbiota development

**Fig. 6:**
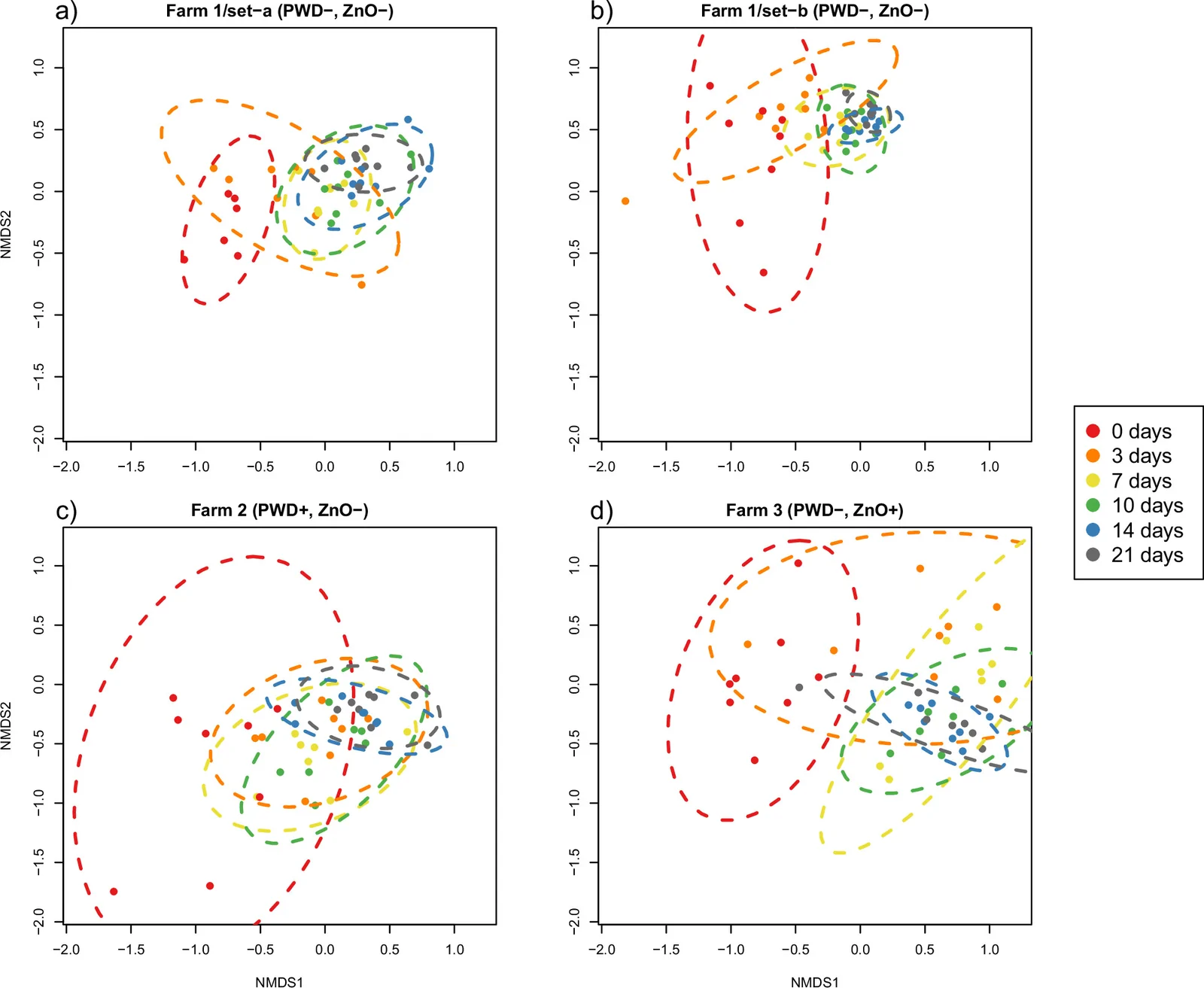
Non-metric multidimensional scaling (NMDS) representation of the microbiota conformation based on the Bray-Curtis distance (Stress = 0.22) from faecal swab samples collected from 32 healthy piglets from three different farms before and after weaning. The gut microbiota conformation was obtained by 16S rRNA gene amplicon sequencing of the V3-V4 regions. Panel **a**) shows the farm without problems with post-weaning diarrhoea (Farm 1/set-a PWD-, ZnO-) and panel **b**) shows the same farm three months later (Farm 1/set-b). Panel **c**) shows the results from the farm with intermittent problems with post-weaning diarrhoea (Farm 2 PWD + , ZnO-) and panel **d**) shows the results from the farm using 2500 ppm of zinc oxide in the feed for 14 days after weaning (Farm 3 PWD-, ZnO + )

In Fig. 6, a non-metric, multidimensional scaling (NMDS) plot is presented, utilising the Bray-Curtis dissimilarity index, based on relative abundances, to visualise the microbiota of each pig in two dimensions. The microbiota of individual pigs on days 0 and 3 were more dispersed compared to subsequent days across all study groups. From day 7 onwards, also including day 3 for Farm 2 (PWD + , ZnO-), ​the microbiota profiles of the pigs began to converge towards a progressively similar state in each study group. This trend was particularly evident for both study groups in Farm 1 (PWD-, ZnO-) and in Farm 2 (PWD + , ZnO-). In Farm 3 (PWD-, ZnO + ), this convergence was less pronounced, with a minor expansion on day 21 compared to day 14. When data from all study groups were combined, there was a decreased dispersion over time (*P* < 0.001). This indicated a progressive shift toward increasingly similar microbiota composition across all groups (Supplementary material, Fig. 2). PERMANOVA based on Bray–Curtis dissimilarities showed significant effects of days after weaning, study group, and their interaction on microbial community composition. Study group explained the largest proportion of the variation (R² = 0.156, *F* = 13.65, *P* = 0.001), while days after weaning accounted for 10.9% of the variation (R² = 0.109, *F* = 5.74, *P* = 0.001). The interaction between study group and days after weaning was also significant (R² = 0.118, *F* = 2.06, *P* = 0.001), indicating that changes in microbial community composition over time differed among study groups. Collectively, days after weaning, study group, and their interaction explained 38.3% of the variation in microbial community composition. These findings were consistent with the patterns observed in the NMDS ordination plots.

## Discussion

In commercial pig production, weaning is one of the most stressful events in a pig’s life. It is well known that this abrupt change leads to a shift in the intestinal microbiota composition [22]. However, the prevalence of PWD and the subsequent need for medications such as high-dose zinc oxide (ZnO) and antibiotics vary widely among farms [47–49]. ZnO has been used extensively to prevent PWD, and after the ban, there has been an increasing need for enhanced understanding of the role of the microbiota in association with weaning. Thus, there is a need to investigate whether differences between farms are associated with the post-weaning development of the intestinal microbiota. An observational cohort study was conducted to compare the faecal microbiota of healthy piglets across three farms, in relation to their difference in the use of ZnO and in their previous records of PWD.

Most commonly after weaning, the relative abundance of bacteria from the phylum Firmicutes increases, and that of Bacteroidota decreases [5, 50]. This shift in the Firmicutes:Bacteroidota ratio, with a relative increase in Firmicutes, was observed in all study groups, except for Farm 1/set-b (PWD-, ZnO-). In that study group, the relative abundance of Bacteroidota instead increased post-weaning, similar to other studies [51]. The differences between studies can be attributed to many factors, such as the use of growth-promoting antibiotics, different weaning ages, availability of straw or other rooting/bedding materials, and genetics. Our findings highlight the difficulty to predetermine the response of the microbiota to factors that influence the gut environment. For Farm 1/set-b (PWD-, ZnO-), the favourable gut environment for bacteria within Bacteroidota could have been influenced by both internal and external factors, but most likely it was dependent on variations in the feed to both sows and piglets since feed producers only have to account for the protein and energy content and not ratios between different types of grains. The provision of straw as rooting and bedding material could also have exerted an influence due to different harvests of straw.

Consistent with findings from other studies, Proteobacteria decreased in relative abundance post-weaning in all study groups [52]. However, Farm 2 (PWD + ,​ZnO​-) had a significantly higher relative abundance of Proteobacteria, which persisted longer during the measurement period compared to the other farms. Proteobacteria in higher abundance have been detected in association with enteric infections and are suggested to be a potential indicator for dysbiosis [28, 53, 54]. It is notable that *E. coli*, which is strongly associated with PWD [17], is a member of this phylum. Consequently, these findings were consistent with the intermittent occurrence of PWD in Farm 2 (PWD + , ZnO-).

Across all farms, the contribution of Actinobacteriota was small and remained so throughout the study period. Notably, the relative abundance was significantly lower in the study groups from Farm 1 (PWD-, ZnO-), compared to Farm 2 (PWD + , ZnO-) and Farm 3 (PWD-, ZnO + ). In addition, the relative abundance of Actinobacteriota increased on Farms 2 and 3 to its highest value on day 10 (Farm 2) and day 21 (Farm 3). The results obtained in Farms 2 and 3 differed from other reported findings that show a decrease in Actinobacteriota post-weaning [55]. This could reflect that the microbiota remained immature for longer post-weaning, as members of Actinobacteriota, especially *Bifidobacterium*, mainly digest simple carbohydrates that are often present in milk [56]. These findings may have indicated a lower intake of complex carbohydrates and may also have been affected by the change of feed composition and intake post-weaning, e.g., the introduction of ZnO at weaning in Farm 3 (PWD-, ZnO + ).

Verrucomicrobiota is a health marker for the gut microbiota, dominated by the genus *Akkermansia* [57]. Being mucin-degrading specialists, an increase is generally seen later in the post-weaning period when mucus production increases due to the change in diet [28]. In this study, the relative abundance was highest in Farm 2 (PWD + , ZnO-), which could have indicated a higher mucus production. The higher relative abundance of Verrucomicrobiota may have contributed to the current healthy status of the sampled piglets on Farm 2 (PWD + , ZnO-) that otherwise had intermittent problems with PWD.

Pre-weaning, a higher relative abundance of Fusobacteriota is generally found compared to after weaning [58], which was consistent with the results in this study. The proportion of Fusobacteriota was less than 5% at weaning and decreased thereafter in all study groups. Also, the proportion of Desulfobacterota was generally low and the phylum was totally absent from three samples. *L. intracellullaris*, causing proliferative enteropathy (PE), belongs to this phylum, which could have explained the absence of clinical signs. However, PE usually occurs from two weeks after weaning, and this study covered the first three weeks [59]. It is also noteworthy that piglets in Farm 3 (PWD-, ZnO + ) were vaccinated against *L. intracellularis*, which contributed to the low abundance in that study group.

The phylum Spirochaetota includes the large intestine pathogens *B. hyodysenteriae* and *B. pilosicoli* in the family Brachyspiraceae [27]. Also, genus *Treponema* in the family Spirochaetaceae, a biomarker of gut microbiota associated with diarrhoea in piglets, belongs to this phylum [26]. Although the proportion of Spirochaetota was low at weaning, it was higher in Farm 2 (PWD + , ZnO-) and Farm 3 (PWD-, ZnO + ) than in both study groups from Farm 1 (PWD-, ZnO-). Post-weaning, the proportion of Spirochaetota decreased in all study groups with the exception of Farm 1/set-b (PWD-, ZnO-), where the proportion somewhat increased on day 3 post-weaning. That observation contradicted the other results and may have indicated an increased risk for diarrhoea. However, after this transient increase, the proportion of Spirochaetota decreased also in this study group, although remaining at a higher level than in the other groups.

The increase seen in Campylobacterota in Farm 1/set-b (PWD-, ZnO-) on day 3 was due to a transient increase in a single healthy pig, indicating that the pig had been exposed to bacteria of that phylum but without persistent colonization.

The higher Bacteroidota relative abundance found in Farm 1/set-b (PWD-, ZnO-), mentioned previously, was mainly represented by bacteria from the family Prevotellaceae. It is common that bacteria in the family Prevotellaceae, and particularly genus *Prevotella*, increase in relative abundance after weaning due to their ability to ferment complex carbohydrates [13, 22, 60]. There were no apparent factors to explain why Farm 1/set-b showed a different pattern of microbiota development, but the higher abundance could reflect a greater dietary fibre intake. A higher fibre content has been shown to promote a higher relative abundance of *Prevotella*, and these bacteria produce short-chain fatty acids (SCFA) that promote gut health [61].

The Proteobacteria family Enterobacteriaceae often decreases in relative abundance after weaning in healthy pigs [22], which was seen in all study groups. However, in Farm 2 (PWD + , ZnO-), there was a greater variation, which could have been consistent with the intermittent presentation of PWD in the herd. Considering that the main PWD-associated bacterium is *E. coli*, belonging to this family, the decline in relative abundance was consistent with the fact that the sampled pigs in the study were healthy. Another Proteobacteria family is Pasteurellaceae, which includes several opportunistic pathogens and is known for causing respiratory disease in pigs by disrupting the mucosal barrier in the airways [62]. Pasteurellaceae was more pronounced in Farm 2 (PWD + , ZnO-) compared to the other study groups, especially on day 10. Higher relative abundance of Pasteurellaceae in faecal samples has been found in human patients with the inflammatory bowel disease Crohn’s disease, as well as in pigs with diarrhoea [63, 64]. This could indicate an association with a disrupted microbiota, consistent with the farm history of recurring PWD problems. The shift from Pasteurellaceae to the T34 family group as the predominant Proteobacteria-associated family between days 10 and 14 in Farm 2 (PWD + , ZnO-), may also reflect an alteration of the intestinal microbial ecosystem coinciding with the intermittent PWD problems. This interpretation is supported by a recent study reporting significantly higher abundance of T34 in diarrheic compared with healthy post-weaning piglets, as well as an increase in T34 abundance over time in diarrheic animals [65]. However, this association should be interpreted cautiously, as T34 represents an uncultured and poorly characterized lineage within the Burkholderiales order, and its specific role in the porcine gut remains largely unknown.

Different families within the phylum Firmicutes respond differently to changes in the diet and environment at weaning, corresponding to their respective niches in the microbiota ecosystem of the gastrointestinal tract (GIT). The *Lactobacillus* genus, being a core component of the porcine gut microbiota [13, 22, 24], was the predominant member of the Lactobacillaceae family in the phylum Firmicutes on each farm. Fermenting carbohydrates into lactic acid, helping maintain an acidic intestinal environment, and inhibiting the growth of potential pathogens, the *Lactobacillus* genus contributes to general gut health and microbial stability [21, 50]. Overall, the fibre-fermenting, butyrate-producing bacterial families Lachnospiraceae and Ruminococcaceae, also from the phylum Firmicutes, increased in relative abundance after weaning. This was in accordance with multiple other studies and reflected a maturing microbiota after weaning [24, 58, 61].

Similar to the other Firmicutes, Erysipelotrichaceae, often increase after weaning [66, 67]. However, findings in some studies suggest that this increase could be a sign of a transient dysbiosis [66], while others associate a higher abundance with improved growth performance [68]. The significantly higher relative abundance of Erysipelotrichaceae in pigs from Farm 2 (PWD + , ZnO-) may have been a response from the gut to the complex carbohydrates in the feed but could also have been a sign of gut disturbance. From current literature, it is not possible to associate a higher relative abundance of Erysipelotrichaeceae with a higher risk for gastrointestinal disease, although some species within Erysipelotrichaceae are associated with other disease symptoms, e.g. *Erysipelothrix rhusopathiae* [69].

Consistent with other studies, the alpha diversity increased after weaning in three of the four study groups. The increase in alpha diversity after weaning is attributed to the change in feed from milk to a grain-based diet [13, 22, 51]. In contrast to the other study groups, the average Shannon diversity in Farm 3 (PWD-, ZnO + ) decreased after weaning. This was likely associated with the use of high-dose ZnO in this herd and/or the underlying factors associated with the occurrence of PWD that led to the use of ZnO. A prominent decrease in Shannon diversity at day 21 was seen for this study group. This could have been due to the discontinuation of ZnO in the feed as well as a change in the feed recipe 14 days post-weaning, creating a need for the microbiota to adapt once again. Still, feeding high-dose ZnO has been shown to stabilise the diversity of the coliform faecal microflora after weaning [25].

The NMDS and PERMANOVA results showed greater dissimilarity of the faecal microbiota between pigs before weaning compared to on days 14 and 21, when the microbiota is assumed to have adapted to the new feed. The individual variation between pigs was large at weaning except for Farm 1/set-a (PWD-, ZnO-). On day 3 after weaning, a prominent change in the microbiota was seen for all farms, which extended into day 7 after weaning for Farm 3 (PWD-, ZnO + ). This pattern was likely an effect of the change in diet. From day 10 and onward, the samples were distinctly clustered together, forming a progressively more similar microbiota across the piglets on each farm, as also shown in previous studies [22, 24]. This indicated that the pigs arrived at the weaning unit with a microbiota originating from the sow/litter environment, which gradually became more diverse in the individual, but between pigs, a more similar microbiota was established. In addition, when the NMDS data for the farms were combined, it was also apparent that the microbiota became more similar between the farms during the post-weaning period.

In this study, loose faeces were observed in a few piglets in the herd without any clinical PWD. Over the study period, the piglets did not develop diarrhoea and the faecal consistency returned to normal. From this observation, it was apparent that weaning is a strain on pigs’ intestinal health, even in herds with well-functioning weaning management, consistent with other studies [6].

Pigs are outbred, and the genetic differences between litters are considerable [70]. Hence, these differences are difficult to compensate for within and between study groups. However, to ameliorate this, piglets from different litters and batches in the same herd were sampled. Despite this, differences were observed between study groups. The considerable difference between samples indicated a large individual variation in the microbiota composition. A larger sample size would reduce the influence of the individual samples on the results, and additional repeated sampling of the same farm could improve the understanding of external factors influencing the microbiota development.With only three farms included in the current study and that these farms differed regarding PWD problems, feed type (liquid and dry) as well as inclusion of ZnO in the feed, confounding management and environmental factors at the farm level cannot be excluded.

The small intestine is the main site of infection for PWD [15, 17] and would have been preferable to sample. This cannot be obtained under field conditions without sacrificing piglets, which in turn would also have made serial samplings from selected individuals impossible. Therefore, faecal swab samples from the rectum were used. Rectal samples ensured freedom from environmental contamination and have been shown to produce equal 16S rRNA sequencing results compared to faecal samples [71]. However, results for the microbiota composition in these types of samples differ compared to samples from other parts of the gastrointestinal tract [66]. Despite this, the results obtained reflected the changes occurring throughout the GIT and are in line with the reduce and refine principles of using animals in research.

Although DNA is stable, some degradation can occur during storage and extraction. With denoising and removing chimaeras, some PCR and sequencing biases can be reduced, but this does not eliminate all possibilities of spurious sequences. Sequencing the 16S gene limits taxonomic classification on the species level, leading to that known pathogens cannot be determined to be present or absent [72].

This study provided a temporal description of microbiota development during the first three weeks after weaning across different commercial pig farms. The higher abundance of Bacteroidota in Farm 1/set-b (PWD-, ZnO-) compared with the other study groups was reflected in an increase in Prevotellaceae, indicating a more mature microbiota and a favourable gut status for a successful weaning. Also, Farm 1/set-a (PWD-, ZnO-) presented indicators of a healthy microbiota, such as a higher relative abundance of genus *Faecalibacterium*. *Faecalibacterium prauznitzii* is especially correlated with many positive effects on intestinal health [66]. On the other hand, Proteobacteria were high in abundance in Farm 2 (PWD + , ZnO-) after weaning, which could represent a higher risk of developing PWD. The use of ZnO stabilised diversity immediately post-weaning, but after the withdrawal of ZnO, the diversity decreased in the study group Farm 3 (PWD-, ZnO + ). In all, defining a favorable gut microbiota for a successful weaning is challenging. Despite the differences seen between the study groups, no piglet developed clinical PWD. The complex, multifactorial effects of weaning on intestinal health and microbiota highlight the need for further studies.

## Conclusion

The results from this study demonstrated a difference between the gut microbiota of piglets before and after weaning in all study groups. The results also clearly demonstrated that weaning led to an imbalance in the gut microbiota and changes in the faecal consistency, even in piglets without clinical PWD. Firmicutes and Bacteroidota were the two dominant phyla throughout the study period in all study groups. However, there were differences in the faecal microbiota composition between study groups and across the study period. The results could indicate that the intermittent PWD problems in one of the herds might be associated with the higher relative abundance of Proteobacteria in that herd. The use of ZnO seemed to have delayed the maturation of the microbiota, although further studies are needed to confirm this. Considering the literature and the results from this study, no single microbiota composition can predict weaning without gut disturbances.

## Supplementary information

Supplementary material_fig_1

Supplementary material_fig_2

## Data Availability

Sequencing data were deposited into the BioProject database under accession number PRJNA1444071 and are available at the following URL: https://www.ncbi.nlm.nih.gov/bioproject/1444071.
