# Supplementary material for: A cohort study of the bacterial faecal microbiota development post-weaning in healthy piglets in pig herds with different intestinal health status
Source: BMC Microbiol. 2026 Nov 6;26:974. doi: 10.1186/s12866-026-05888-7 (PMC13744104; doi:10.1186/s12866-026-05888-7)
Supplement: Supplementary file 1 [file 12866_2026_5888_MOESM1_ESM.pdf]

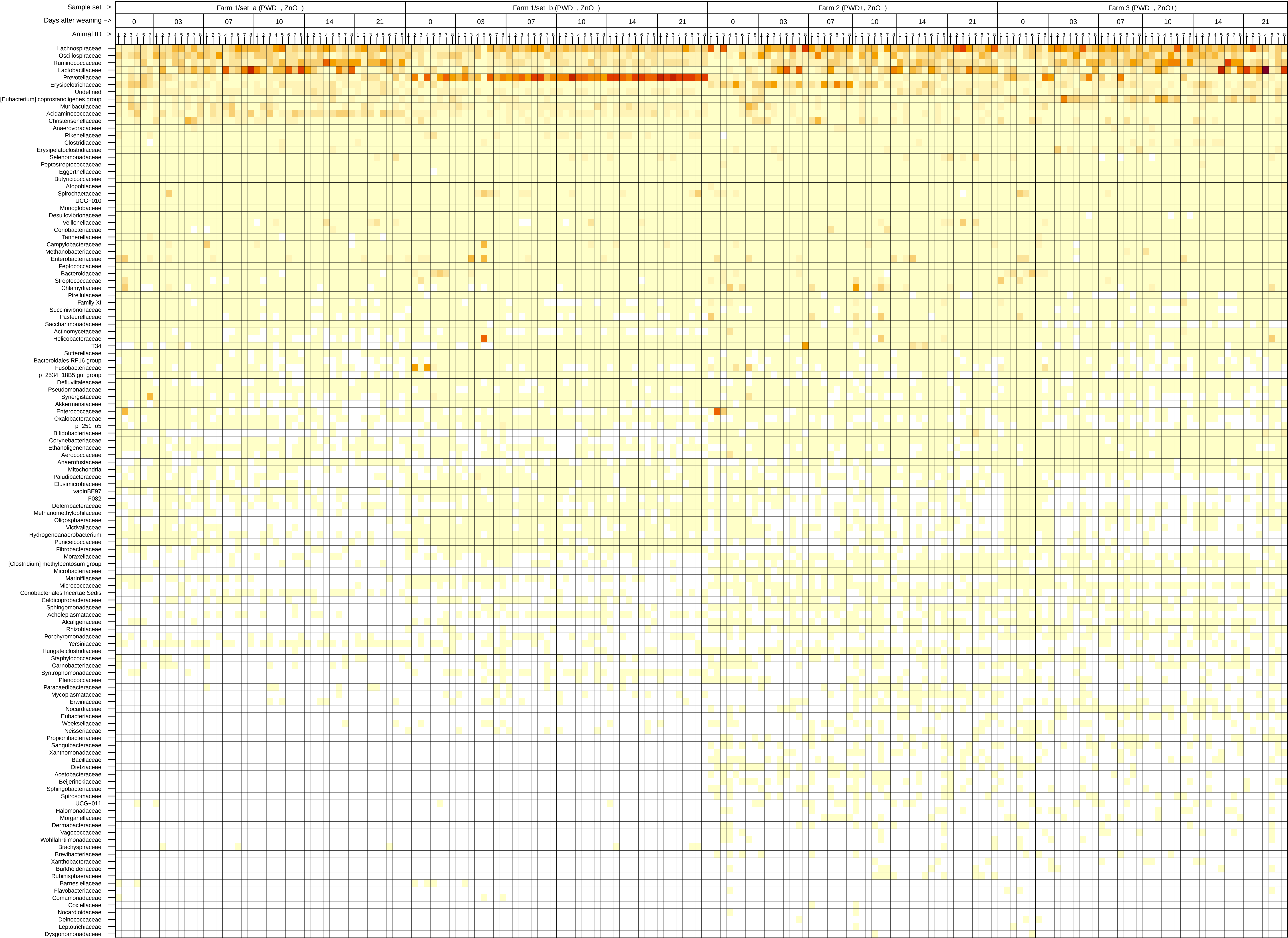
Sample set ->
Farm 1/set−a (PWD−, ZnO−)
Farm 1/set−b (PWD−, ZnO−)
Farm 2 (PWD+, ZnO−)
Farm 3 (PWD−, ZnO+)
Days after weaning ->
0
03
07
10
14
21
Animal ID ->
Lachnospiraceae
Oscillospiraceae
Ruminococcaceae
Lactobacillaceae
Prevotellaceae
Erysipelotrichaceae
Undefined
[Eubacterium] coprostanoligenes group
Muribaculaceae
Acidaminococcaceae
Christensenellaceae
Anaerovoracaceae
Rikenellaceae
Clostridiaceae
Erysipelatoclostridiaceae
Selenomonadaceae
Peptostreptococcaceae
Eggerthellaceae
Butyricicoccaceae
Atopobiaceae
Spirochaetaceae
UCG−010
Monoglobaceae
Desulfovibrionaceae
Veillonellaceae
Coriobacteriaceae
Tannerellaceae
Campylobacteraceae
Methanobacteriaceae
Enterobacteriaceae
Peptococcaceae
Bacteroidaceae
Streptococcaceae
Chlamydiaceae
Pirellulaceae
Family XI
Succinivibrionaceae
Pasteurellaceae
Saccharimonadaceae
Actinomycetaceae
Helicobacteraceae
T34
Sutterellaceae
Bacteroidales RF16 group
Fusobacteriaceae
p−2534−18B5 gut group
Defluviitaleaceae
Pseudomonadaceae
Synergistaceae
Akkermansiaceae
Enterococcaceae
Oxalobacteraceae
p−251−o5
Bifidobacteriaceae
Corynebacteriaceae
Ethanoligenenaceae
Aerococcaceae
Anaerofustaceae
Mitochondria
Paludibacteraceae
Elusimicrobiaceae
vadinBE97
F082
Deferribacteraceae
Methanomethylophilaceae
Oligosphaeraceae
Victivallaceae
Hydrogenoanaerobacterium
Puniceicoccaceae
Fibrobacteraceae
Moraxellaceae
[Clostridium] methylpentosum group
Microbacteriaceae
Marinifilaceae
Micrococcaceae
Coriobacteriales Incertae Sedis
Caldicoprobacteraceae
Sphingomonadaceae
Acholeplasmataceae
Alcaligenaceae
Rhizobiaceae
Porphyromonadaceae
Yersiniaceae
Hungateiclostridiaceae
Staphylococcaceae
Carnobacteriaceae
Syntrophomonadaceae
Planococcaceae
Paracaedibacteraceae
Mycoplasmataceae
Erwiniaceae
Nocardiaceae
Eubacteriaceae
Weeksellaceae
Neisseriaceae
Propionibacteriaceae
Sanguibacteraceae
Xanthomonadaceae
Bacillaceae
Dietziaceae
Acetobacteraceae
Beijerinckiaceae
Sphingobacteriaceae
Spirosomaceae
UCG−011
Halomonadaceae
Morganellaceae
Dermabacteraceae
Vagococcaceae
Wohlfahrtiimonadaceae
Brachyspiraceae
Brevibacteriaceae
Xanthobacteraceae
Burkholderiaceae
Rubinisphaeraceae
Barnesiellaceae
Flavobacteriaceae
Comamonadaceae
Coxiellaceae
Nocardioidaceae
Deinococcaceae
Leptotrichiaceae
Dysgonomonadaceae

Suppl. Fig. 1

Heatmap of faecal microbiota family composition across individual pig samples from faecal swab samples collected from 32 healthy piglets from three different farms before and after weaning. Each column represents a single pig sample, and each row corresponds to a bacterial family identified within the dataset. Color intensity reflects the relative abundance of each family, with more intense color indicating higher abundance. The gut microbiota conformation was obtained by 16S rRNA gene amplicon sequencing of the V3-V4 regions.
