# Supplementary material for: A cohort study of the bacterial faecal microbiota development post-weaning in healthy piglets in pig herds with different intestinal health status
Source: BMC Microbiol. 2026 Nov 6;26:974. doi: 10.1186/s12866-026-05888-7 (PMC13744104; doi:10.1186/s12866-026-05888-7)
Supplement: Supplementary file 2 [file 12866_2026_5888_MOESM2_ESM.pdf]

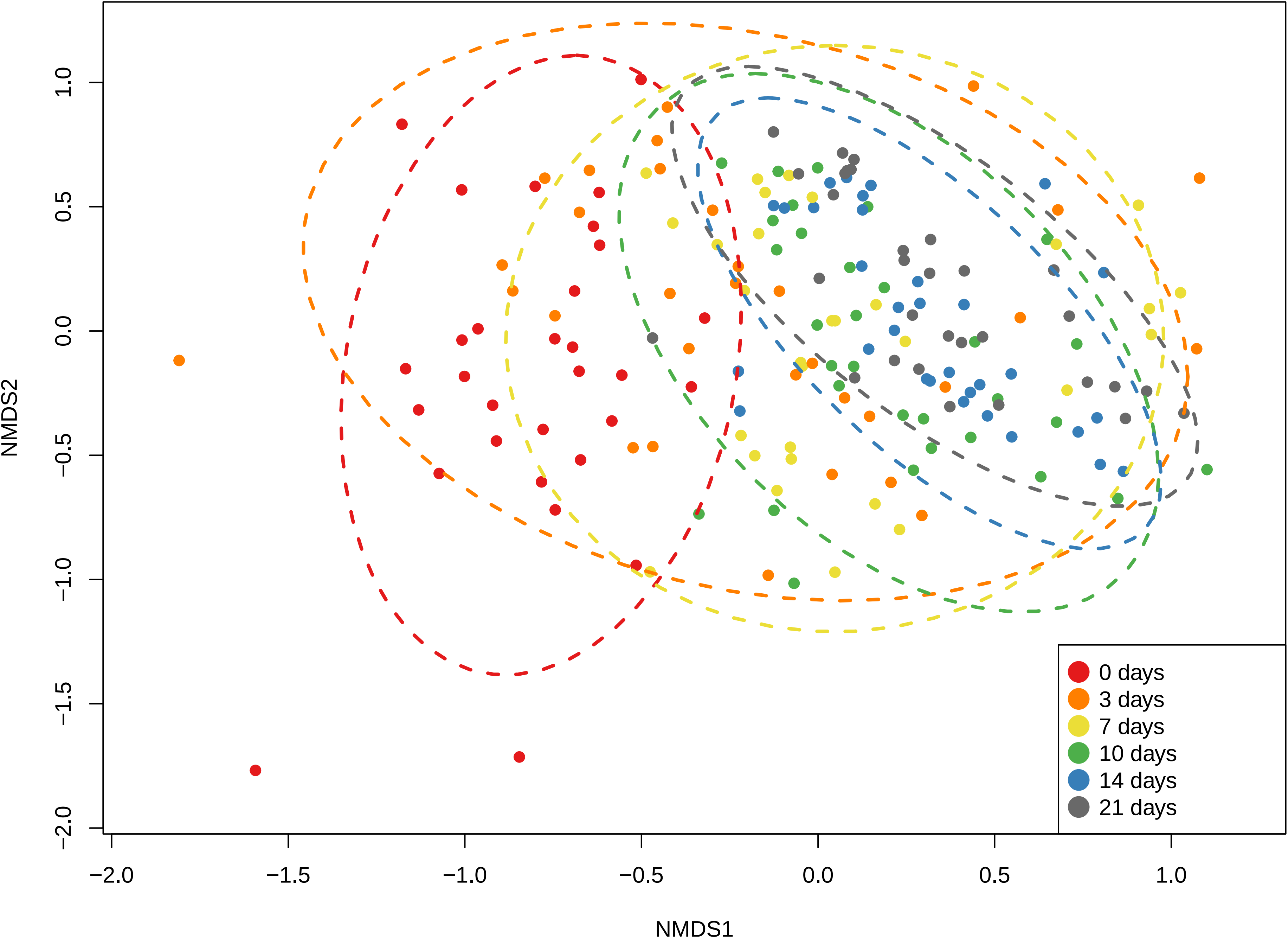

NMDS1
NMDS2
−2.0
−1.5
−1.0
−0.5
0.0
0.5
1.0
0 days
3 days
7 days
10 days
14 days
21 days

Suppl. Fig. 2

Non-metric multidimensional scaling (NMDS) representation of the microbiota conformation based on the Bray-Curtis distance (Stress= 0.22) from faecal swab samples collected from 32 healthy piglets from three different farms before and after weaning. The gut microbiota conformation was obtained by 16S rRNA gene amplicon sequencing of the V3-V4 regions.
